# Profil étiologique des surdités neurosensorielle sévère et profonde de l'enfant dans la région du centre-nord du Maroc

**DOI:** 10.11604/pamj.2014.17.100.2331

**Published:** 2014-02-08

**Authors:** Mohammed Ridal, Naouar Outtasi, Zainab Taybi, Redouan Boulouiz, Sanae Chaouki, Meryem Boubou, Mustapha Maaroufi, Najib Benmansour, Zouheir Zaki, Karim Ouldim, Hamid Barakat, Mustapha Hida, Siham Tizniti, Mohamed Noreddine El Alami

**Affiliations:** 1 Service d'Oto-Rhino-Laryngologie, CHU Hassan II Fés et faculté de médecine de Fès, Maroc; 2 Laboratoire de biologie moléculaire, Institut Pasteur Casablanca, Maroc; 3 Service de pédiatrie. CHU Hassan II Fès, Maroc; 4 Service de Radiologie, CHU Hassan II, Maroc; 5 Laboraoire de génétique médicale, CHU Hassan II Fès, Maroc

**Keywords:** Surdité neurosensorielle, génétique, méningite, Maroc, sensorineural deafness, genetic, meningitis, Morocco

## Abstract

**Introduction:**

Le diagnostic d'une surdité profonde est possible dès les premiers jours de vie. Or, le développement du langage et l'intégration scolaire et professionnelle ne sont pas possible que si la surdité est prise en charge précocement. L’établissement d'un diagnostc étiologique a des implications pronostiques et thérapeutiques.

**Méthodes:**

C'est une étude rétrospective allant de Juin 2009 au mois de Janvier 2012 ayant recensé 250 cas d'enfants porteurs d'une surdité sévère et profonde.

**Résultats:**

La moyenne d’âge au moment de l'annonce du diagnostic est de 3.7 ans. Les étiologies prédominantes sont les surdités génétiques dans 35.6% suivies des surdités acquises dans 30.8% des cas. Dans 34.4% des cas aucune étiologie n'a pu être retrouvée.

**Conclusion:**

Cette étude met en évidence la prédominance éventuelle de causes génétiques de la surdité neurosensorielle de l'enfant au Maroc, et souligne la nécessité d'améliorer les politiques de prévention des maladies infectieuses et de dépistage de la surdité néonatale. Cependant, des analyses moléculaires plus ciblées et la réalisation d'un scanner des rochers systématiques sont nécessaires pour évaluer plus précisément la contribution des étiologies génétiques.

## Introduction

La surdité est le déficit sensoriel le plus fréquent chez l'enfant [1]. Son retentissement socio-économique justifie la nécessité d'un diagnostic précoce. Ce dernier fait appel à un dépistage ciblé ou systématique, un bilan audiologique et étiologique et une prise en charge à la fois prothétique et orthophonique [2]. La sévérité de la surdité influence considérablement les répercussions sur le langage et donc l’âge de suspicion. Le degré de surdité est établi en fonction des seuils d'audition mesurés par l'audiométrie tonale, selon les critères établis parle Bureau International d'Audiophonologie [3]: on calcule la moyenne des seuils sur les fréquences 500,1000, 2000 et 4000 Hz, sur la meilleure oreille. La surdité sévère signifie une perte moyenne de plus de 70 dB alors que dans une profonde elle est de 90 dB. L′âge moyen de diagnostic est de 7 mois pour la surdité profonde et 11 mois et de 17 mois pour la surdité sévère en dehors d′un dépistage universel [4]. De ce fait le diagnostic d'une surdité sévère et profonde de l'enfant est toujours une urgence.

## Méthodes

C′est une étude rétrospective allant de Juin 2009 au mois de Janvier 2012 ayant recensé 250 cas d'enfants porteurs d'une surdité sévère ou profonde. Nos patients ont bénéficié d'un examen clinique, une exploration fonctionnelle de l'audition, une imagerie, une consultation de génétique (pour les surdités congénitales) systématiques. Les autres consultations étaient demandées en fonction des signes d'appel. Les surdités post otitiques (Otite externe, otite séro-muqueuse, otite moyenne aigue et otite moyenne chronique) ont été exclu de ce travail.

Le bilan étiologique commence d′abord par un interrogatoire minutieux sur le déroulement de la grossesse et de l′accouchement à la recherche des facteurs favorisant la surdité pré ou néonatale. Les antécédents de prise médicamenteuse ototoxique, de méningite, de fracture des rochers, de chirurgie de l′oreille et les antécédents familiaux de surdité sont recherchés. Les étapes de développement psychomoteur et du langage ainsi que le statut vaccinal de l′enfant sont notés sur le dossier. L′examen clinique recherche une malformation des oreilles et les autres malformations cranio-faciales. Devant une surdité de l′enfant sans causes acquises évidente une consultation génétique à la recherche d′une mutation causale de la surdité, une tomodensitométrie des rochers à la recherche des malformations de l′oreille externe, moyenne et interne. L′ECG (allongement de l′espace QT dans le syndrome de Jervell Lange Nielsen) et la recherche d′une hématurie par bandelette urinaire en cas de surdité évolutive (syndrome d′Alport) sont systématiques. Le reste du bilan est fonction des cas cliniques et de pathologies associées.

## Résultats

**Figure 1:**
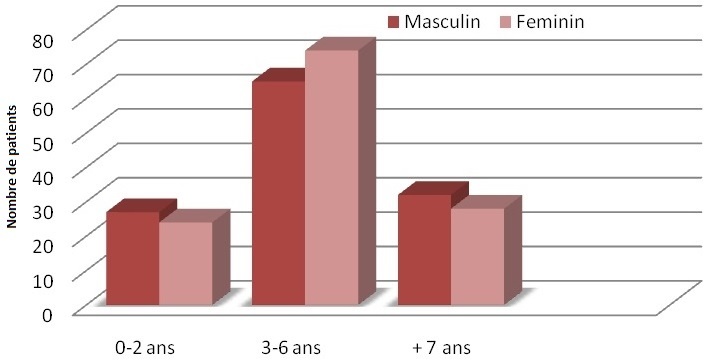
Age moyen de diagnostic de la surdité neurosensorielle

250 enfants répartis en 53.2% (n=133) de sexe féminin et 46.8% (n=117) de sexe masculin sont inclus dans ce travail. La médiane d′âge au moment d′annonce du diagnostic est 3,7 ans avec des extrêmes allant de 4 mois à 16 ans (Figure 1).

Le retard d′acquisition du langage de l′enfant et les troubles de comportement rapportés par les parents, sont les motifs de consultation les plus fréquents (210 cas soit 84%). Les autres consultations étaient demandées par des pédiatres ou bien des ORL pour suspiscion de surdité.

L′origine des enfants selon les villes et régions est comme suit: Fès 118 cas, Taounate 45 cas, Immouzerkandar 32 cas, Taza 22 cas, Meknès 13 cas Errachidia 11 cas, Oujda 6 cas, Khmissat 1 cas, Rabat 1 cas et Azrou 1 cas. Le mariage consanguin parental est retrouvé dans 32% (n=80), essentiellement pour les parents habitant dans les régions rurales. Les antécédents familiaux de surdité sont présents dans 12% des familles (n=30). Le suivi médical de la grossesse n′est renseigné que dans 60% des cas. L′accouchement était dans un milieu médicalisé dans 80% et 25% des cas pour les enfants originaires des milieux urbain et rural respectivement. Le droulement de la grossesse était pathologique dans 4% (n=10) avec 6 infections urinaires traitées avec des médicaments ototoxiques et 4 retards de croissance intra-utérine dont la cause reste indétérminée. 8% des naissances (n=20) ont eu un séjour en réanimation néonatale avec 7 cas de souffrance néonatale, 5 cas d′ictère néonatal ayant nécéssité une exsanguino-transfusion 5 cas d′infection néonatale sévère traitée avec des médicaments ototoxiques et 3 cas de prématurité avec un âge gestastionnel de 33 semaines. La méningite est retrouvée dans 16% des cas (n=40) avec 29 cas à pneucoque, 9 cas à méningocoque et 2 cas à Hémophilus influenzae. 5 enfants (2%) avaient des antécédants de traiement par des médicaments ototoxiques. Un traumatisme crânien occasionnant une fracture du rocher associée à une surdité profonde homalatérale, suite à un accident de la voie publique est retrouvé dans 2 cas (soit 0.8%). En résumé les surdités acquises représentent 30.8% des cas (n=77) avec 4% de causes prénatales, 8% de causes périnatales et 18.8% de causes post-natales.

Le bilan audiologique trouve: une surdité bilatérale dans 96% des cas (n=240) et unilatérale dans 4% des cas (n=10). La surdité est profonde dans 86% des cas (n=215) et sévère dans 14% des cas (n=25). La tomodensitométrie des rochers faite dans 52% des cas (n=130) objective dans 35 cas une labyrinthite ossifiante, dans 14 cas une dilatation de l′acqueduc du vestibule et dans 3 cas une malformation de Mondini. Aucune sérologie de Cytomégalovirus n′est demandée.

Les surdités génétiques sont retrouvées dans 35.6% des cas (n=89):

Les surdités génétiques isolées retrouvées dans 31.6% des cas ( n=79): les surdités autosomiques récessives sont prédominantes et représentées essentiellement par la mutation 35 delG de la connexine 26 retrouvée dans 28% des cas (70/250) dont 82.9% (n=58) à l′état homozygote et 17.1% (n=12) à l′état hétérozygote. Les autres mutations 3.6% (n=9) sont représentés dans le Tableau 1. Aucune mutation du gène GJB6 n′a été retrouvée.

**Tableau 1:** Fréquence des mutations des surdités génétiques isolées par rapport au total des surdités congénitales

Génotype	Nombre de muation	Pourcentage (%) / surdités congénitales (n=173)
**35delG/35delG**	58	33.5
**E47X/35delG**	5	2.9
**35delG/wt**	7	4.1
**V371/wt**	9	5.2
**Total**	79	45.7

Les surdités génétiques syndromiques retrouvées dan 4% des cas (n=10): 6 cas de syndrome de Pendred (2.4%) chez 2 sœurs jumelles et 3 filles et un garçon de familles différentes présentant une surdité sévères bilatérales avec un goitre et une dilatation de l′aqueduc du vestibule sur le scanner des rochers *2 garçons de 11ans et 14 ans présentent un syndrome d′Alport (0.8%) suivis pour une insuffisance rénale débutante et dont l′audiométrie révèle une surdité sévère bilatérale évolutive; deux frères (0.8%) de 14 ans et 16 ans issus d′un mariage consanguin présentent un syndrome de Bardet et Biedel avec une surdité sévère bilatérale évolutive, une rétinite pigmentaire et microphtalmie bilatérale, une gynécomastie, un diabète type II, un hypogonadisme hypergonadotrope et une bradydactylie.

Dans 33.6% des cas soit 84 cas de surdité l′étiologie n′est pas encore déterminée. Les différents résultats sont représentés dans le Tableau 2.

**Tableau 2:** Proportion des étiologies des surdités neurosensorielles de 250 enfants

	Nombre	Pourcentage
Surdités acquise	77	30.8%
		causes prénatales 4%
		causes périnatales 8%
		causes post-natales18,8%
Surdités génétiques	89	35.6%
		Isolées 31.6%
		Syndromiques 4%
Etiologie indétérminée	84	33.6%
Total	250	100

## Discussion

La surdité est le déficit neurosensoriel le plus fréquent chez l′enfant. La prévalence des surdités bilatérales sévères et profondes est estimée entre 0.5 à deux enfants pour 1000 naissances [1, 2]. Au Maroc on compte environ 640000 naissances par an [5] ce qui correspondrait à 640 nouveau-nés qui naissent avec une surdité bilatérale sévère ou profonde par an sans énumérer les surdités acquises. Un autre enfant sur mille devient sourd profond avant l′âge adulte [6]. Ces chiffres démontrent bien que la surdité de l′enfant doit être considérée comme un problème de la santé publique au Maroc. De nos jours, au Maroc, plusieurs équipes hospitalières sont entrain d′instaurer un dépistage systématique universel de la surdité.

En l′absence d′un dépistage de la surdité, cette dernière doit être évoquée devant [7]: des antécédents familiaux de surdité, l'absence de réaction aux bruits ou d'orientation versla source sonore (à partir de 6 mois), une absence ou un retard d'apparition du langage, un langage qui se dégrade (en faveur d'une surdité évolutive), des troubles du comportement (agressivité, anxiété, apathie), un doute des parents.

Le diagnostic de surdité doit alors être posé le plus rapidement possible et avec précision pour permettre une prise en charge adaptée et la plus précoce possible. Il repose essentiellement surla réalisation d'une audiométrie comportementale.

Selon la littérature, environ 50% des surdités neurosensorielles de l′enfant sont d'origine génétique [1, 8], contre 34.8% dans notre série, environ 25% des surdités sont d'origine acquise contre 30.8% dans notre série et environ 25% restent d′origine inconnue contre 34.4% dans notre série. Les surdités génétiques peuvent être congénitales ou d'apparition post-natale. Les surdités acquises peuvent s'installer en période pré, péri ou post-natales.

Parmi ces surdités génétiques, 30% sont associées à d'autres symptômes ou malformations: elles sont alors dîtes syndromiques [6]. Dans les 70% restants, la surdité est le seul symptôme: elle est dite isolée. Ces surdités isolées sont classées selon leur mode de transmission [9]: **les surdités autosomiques récessives ou DFNB (deafness B):** sont les plus fréquentes représentent 70% à 80% des cas de surdité génétiques de l′enfant [10]. La surdité est congénitale, profonde et peu évolutive. Les deux parents hétérozygotes sont normoentendants mais présentent chacun une mutation sur le gène et peuvent le transmettre aussi bien à leurs filles qu’à leurs fils.

La mutation d′un gène occupant le locus DFNB1 est responsable de plus 50% des surdités autosomiques récessives [4]: le GJB2 codant la connexine 26. Cette dernière joue un rôle important dans la genèse du potentiel endocochléaire. La proportion de la mutation 35delG, par rapport à d′autres, du gène GJB2 prédomine largement dans les pays méditerranéens et représente 80% des cas [11–17]. La mutation 35delG homozygote représente la cause de 33.5% des surdités congénitales dans notre série et ressemble aux résultats d′autres pays méditerranéens (Tableau 3).

**Tableau 3:** La prévalence de la mutation du GJB2 et la 35delG/35delG dans le pourtour méditerranéen

Pays	Patients (n)	GJB2 (%)	35delG/35delG (%)	Références
France	88	39.8	28.4	11
Italie/Espagne	136	36.8	32.4	12
Grèce	210	33.3	30	13
Turquie	60	31.7	21.7	14
Lebanon	48	33.3	31.3	15
Egypte	111	14.4	9	16
Tunisie	102	22.55	20.55	17
Maroc	173	40.4	33.5	Notre série

Dans notre étude 5 patients avaient une mutation E47X/35delG, 7 patients avaient une mutation 35delg/wt et 9 enfants avaient une mutation V371/wt. Ces génotypes ont été décrits dans plusieurs publications de surdités sévères et profondes [18–23]. La mutation du gène GJB6 fréquente en Europe et aux USA [24, 25] n′a pas été retrouvée dans notre série et dans d′autres études faites au Maroc [26].

Les surdités autosomiques dominantes ou DFNA (deafness A) représentent 12 à 15% des cas des surdités neurosensorielles de l′enfant [10, 27]. Un des parents sourds transmet l′allèle muté à ses filles ou à ses fils. La surdité est souvent progressive, ou retardée au cours de l'enfance ou à l’âge adulte, et moins sévères [9].

Les surdités liées à l'X ou DFN ne représentent que 1 à 3% des cas de surdité isolée. La mutation est située dans un gène localisé sur le chromosome X. Une mère normoentendante peut transmettre le gène malade à ses fils. La surdité est sévère et profonde [8, 27].

Dix enfants de notre série présentaient une surdité syndromique avec 6 cas de syndrome de Pendred 2 cas de syndrome d′Alport et 2 cas de syndrome de Bardet et Biedel.

Le syndrome de Pendred représente 5% des surdités d'origine génétique pour certains. Il doit être évoqué devant une surdité évolutive, parfois fluctuante. Cette surdité de perception est de transmission autosomique récessive, prélinguale ou postlinguale précoce, bilatérale, profonde, prédominant sur les fréquences aiguës [8]. La surdité associe un goitre qui se développe dans la deuxième décennie et des malformations de l'oreille interne à la tomodensitométrie des rochers (dilatation de l′aqueduc du vestibule, dysplasie de la cochlée à type Mondini). Ce syndrome est dû à des mutations localisées dans le gène PDS ou SLC26A4, localisé sur le chromosome 7 [28].

Le syndrome d′Alport est en général transmis selon le mode dominant lié à l'X et du à des mutations du gène COL4A5 ainsi les garçons et les filles peuvent être atteints. Cependant, les filles sont moins sévèrement atteintes et leur maladie débute plus tardivement. La surdité progressive, qui débute dans la première ou la deuxième décennie (postlinguale) [29], est associée à une atteinte rénale, objectivée initialement par une hématurie, et qui évolue progressivement vers l'insuffisance rénale. La bandelette urinaire systématique chez l'enfant sourd permet un diagnostic et une prise en charge précoces du syndrome d'Alport [4–8].

Le syndrome de Bardet-Biedl, considéré pour longtemps comme est une maladie héréditaire complexe (plus de 17 gènes responsables) et rare. Il touche 1/160000. Il associe une obésité, une rétinite pigmentaire, une hexadactylie postaxiale, un hypogonadisme, une atteinte rénale et un retard mental très variable et souvent modéré. Une grande variété de gènes a été définie; mais seules six qui ont pu être identifiés. Le diagnostic est clinique reposant sur la présence de critères majeurs. La surdité de perception n′est retrouvée que dans 10% des cas [30].

Néanmoins un groupe de surdités reste d’étiologie indéterminée, ceci pourrait être expliqué, hormis le faite d'une enquête étiologique insuffisante, par une hérédité polygénique et multifactorielle ou par une double hétérozygotie. Selon la littérature les causes acquises ne représentent que 25% des surdités de l′enfant [4–32] alors qu′elles représentent 30.8% de l′ensemble des surdités dans notre série.

Pour les surdités acquis es on distingue trois sous groupes: - Les surdités prénatales représentent, dans la littérature, 10% de toutes les surdités neurosensorielles de l'enfant alors que leur taux dans notre série est de 4%. Les causes principales sont les infections du groupe TORSCH (Toxoplasmose, Rubéole, Syphilis, Cytomégalovirus, Herpès), les troubles de développement intra-utérin, le traitement ototoxique au cours de la grossesse [32]. Dans les pays développés l′infection au CMV est en nette augmentation depuis la généralisation du dépistage. L′infection touche jusqu′à 2% des naissances vivantes dans le monde entier, et peut résulter du passage transplacentaire de la primo-infection maternelle. Seulement 5 à 10% des enfants atteints par cette infection ont une surdité neurosensorielle à la naissance et environ 20% avant l′âge de 3 ans [32–34]. Les sérologies CMV n′ont pas été demandées dans notre série. Ceci s′explique par le faible intérêt de cet examen après les premiers six mois de vie. Après ce délai, seule la négativité de ce test présente l′intérêt d′exclure cette étiologie [1].

En raison de le programme de vaccination, les enfants avec déficience auditive due à une infection prénatale de la rubéole sont de plus en plus peu fréquents. L′infection maternelle pendant le premier trimestre peut entraîner, en plus d′une surdité de perception sévère ou profonde bilatérale, un faible poids de naissance, un retard mental, des anomalies cardiaques, une cataracte et une rétinopathie [34].

La toxoplasmose prénatale peut être transmise d′une mère asymptomatique avec une primo-infection, en particulier dans le premier trimestre, et se manifeste classiquement chez le fœtus par une choriorétinite, une hydrocéphalie et des calcifications intracrâniennes. L′association d′une surdité neurosensorielle est rare et peut se présenter plus tard dans l′enfance [34, 35].

La syphilis congénitale est rare (0.002% des naissances vivantes). Elle peut provoquer une surdité neurosensorielle d′installation tardive pouvant aller jusqu′à l′âge de 30 à 40 ans [32].

Les médicaments ototoxiques, l′alcoolisme, les troubles métaboliques et les anomalies de développement de l′oreille interne au cours de la grossesse peuvent être responsables d′une surdité neurosensorielle [34].

Les surdités périnatales représentent environ 15% de l'ensemble des surdités neurosensorielles selon la littérature contre 8% dans notre étude. Plusieurs facteurs sont en cause: le faible poids de naissance (< 1.5 kg), la prématurité avec un âge gestationnel inférieur à 34 semaines, l′hyperbilirubinémie requérant une exsanguino-transfusion, la médication ototoxique, un score d'APGAR <5 à 1 minute, et de <7 à 5 min et une ventilation artificielle pendant 5 jours ou plus [2, 4, 33, 34, 36]. Dans notre étude les antécédents d′une pathologie périnatale causale d′une surdité neurosensorielle sont retrouvés chez 20 enfants.

Les surdités post-natales qui représentent selon la littérature entre 7 à 11% des surdités neurosensorielles de l'enfant, leur taux dans notre étude est de 18%. Elles sont liées à l'administration de médications ototoxiques, aux fractures du rocher (fractures labyrinthiques) et aux méningites bactériennes.

Selon plusieurs auteurs la méningite peut être responsable de plus de 6% des surdités neurosensorielles de l′enfant contre 16% dans notre série. Parmi lesquelles les méningites à pneumocoques comportent un risque de surdité d'environ 30% par rapport à 10% pour les méningites à méningocoques et 6% pour les méningites à Hemophilus influenzae [33, 34]. Ces méningites provoquent des labyrinthites ossifiantes qui peuvent parfois s'installer très rapidement et rendre extrêmement difficile, voire aléatoire, une implantation cochléaire. L′Académie américaine des pédiatres recommande l′association du dexaméthasone dans le traitement des méningites pour diminuer le risque de surdité [36].

Beaucoup d′agents pharmacologiques peuvent avoir des effets toxiques sur l′oreille interne responsables ainsi de surdités neurosensorielles et/ou des troubles de l′équilibre. La liste comprend les aminosides (par exemple la gentamicine, la streptomycine), les produits de la chimiothérapie (par exemple cisplatine), les salicylates, la quinine, et les diurétiques de l′anse [34–38]. L′ototoxicité est généralement associée à une surdité neurosensorielle bilatérale symétrique et peut être temporaire ou irréversible et des acouphènes. La surdité touche d′abord les fréquences aigues puis si le traitement perdure elle concerne toutes les fréquences. L′apparition est imprévisible et peut survenir même après une seule dose, ou après plusieurs semaines. La physiopathologie dépend de l′agent, et comprend la formation de radicaux libres responsables de la destruction des cils des cellules cochléaires, et des dommages au niveau de la strie vasculaire [38, 39]. Les aminosides perturbent la synthèse mitochondriale des protéines surtout pour les enfants porteurs de la mutation de l′ARN mitochondrial 12S rRNA [40, 41].

Autres causes peuvent être responsables de surdités neurosensorielles chez l′enfant en particulier les fractures transversales du rocher translabyrinthique créant une ouverture des cavités labyrinthiques vers l'oreille moyenne avec fistule périlymphatique majeure et apparition d'air intralabyrinthique (pneumolabyrinthe).

Elles sont fréquemment associées à une brèche méningée [42–44]. Ces lésions sont généralement immédiates, définitives et complètes, rarement partielles. Il s'agit d'un grand vertige rotatoire avec nausées et vomissements accompagné d'une surdité de perception sévère (2 surdités profondes dans notre étude). Une paralysie faciale est associée dans 20% des cas [43]. Le bilan tomodensitométrique met en général en évidence le trait de fracture ou un pneumolabyrinthe [42–45].

Dans notre 33.6% des cas (n=84) de surdité l′étiologie n′est pas encore déterminée. Ce chiffre varie dans la littérature entre 25-40% [1, 2, 6, 36]. La différence d′âge de nos patients (avec des extrêmes de 4 mois et 16 ans) peut induire des oublis de la part des parents sur différents éléments cliniques et compromettre la recherche étiologique. Selon les auteurs les infections materno-fœtales sont sous estimés de même que les causes génétiques en raison de gènes non encore identifiés ou non testés systématiquement [1, 46, 47].

## Conclusion

L'enquête étiologique d'une surdité neurosensorielle de l'enfant se base avant tout sur un interrogatoire minutieux systématique, un examen clinique complet et des explorations paracliniques hiérarchisés. Ces dernières années, des progrès significatifs ont été réalisés tant en matière du bilan génétique que sérologique et d'imagerie, permettant ainsi à l'enfant atteint d'une surdité sévère ou profonde une prise en charge adaptée pour atténuer les effets négatifs de la privation auditive au niveau de la structuration du système auditif central et une évolution beaucoup plus facilement dans le monde entendant.
